# The landscape of alternative splicing reveals novel events associated with tumorigenesis and the immune microenvironment in gastric cancer

**DOI:** 10.18632/aging.202393

**Published:** 2021-01-10

**Authors:** Shenghan Lou, Jian Zhang, Zhao Zhai, Xin Yin, Yimin Wang, Tianyi Fang, Yingwei Xue

**Affiliations:** 1 Department of Gastroenterological Surgery, Harbin Medical University Cancer Hospital, 150081 Harbin, Heilongjiang Province, China; 2 Department of Thoracic Surgery, Harbin Medical University Cancer Hospital, 150081 Harbin, Heilongjiang Province, China

**Keywords:** gastric cancer, alternative splicing, RNA-seq profile, tumorigenesis, immune microenvironment

## Abstract

Alternative splicing (AS), contributing to vast protein diversity from a rather limited number of genes in eukaryotic transcripts, has emerged as an important signature for tumor initiation and progression. However, a systematic understanding of its functional impact and relevance to gastric cancer (GC) tumorigenesis is lacking. Differentially expressed AS (DEAS) was verified among GC-associated AS events based on RNA-seq profiles from the TCGA database. Functional enrichment analysis, unsupervised clustering analysis and prognostic models were used to infer the potential roles of DEAS events and their molecular, clinical and immune features. In total, 12,225 AS events were detected from 5,199 genes, among which 314 AS events were identified as DEAS events in GC. The parental genes of the DEAS events were significantly enriched in the regulation of GC-related processes. The splicing correlation network suggested a significant relationship between DEAS events and splicing factors (SFs). Three clusters of DEAS events were identified to be different in prognosis, cancer-specific signatures and immune features between distinct clusters. Univariate and multivariate analyses regarded 3 DEAS events as independent prognostic indicators. Profiling of the AS landscape in GC elucidated the functional roles of the splicing network in GC and might serve as a novel prognostic indicator and therapeutic target.

## INTRODUCTION

With advances in the early screening, diagnosis and treatment of gastric cancer (GC), its annual incidence and mortality have decreased [1]. However, GC is still the fifth most frequently diagnosed cancer and the third leading cause of cancer-related mortality, with almost 1,000,000 new cases and 800,000 deaths each year [2, 3]. Due to indistinctive symptoms, GC often exhibits proliferation, extensive invasion and lymphatic metastasis at the time of diagnosis, contributing to its poor prognosis [4]. The pathogenesis of GC is complex, as it is controlled by genetic alterations in oncogenes and suppressor genes, and these alterations result in disease heterogeneity [4]. Despite advances in the molecular characteristics of GC, the prospect of tailored individual therapy based on histological and molecular subtypes is still unsatisfactory. The overall 5-year relative survival rate of GC patients is still quite low [5]. Therefore, it is worthwhile to explore the novel biological indicators and molecular mechanisms of GC.

Cancer profiling has allowed more dimensionalities due to advances in the depth and quality of transcriptome sequencing in the era of cancer genomics [6]. Alternative splicing (AS) is one of the most crucial posttranscriptional regulatory mechanisms and modifies more than 90% of human genes [7]. AS significantly enriches protein diversity by generating different RNA isoforms of single genes [8]. AS may result in a series of consequences, such as changing the stability of proteins, adding or deleting structural domains and modifying the interactive relationship between proteins [9]. Abnormal AS events participate in several tumorigenic processes, such as proliferation, apoptosis, angiogenesis and metastasis [10]. The initial unbalanced expression of splice isoforms or the accumulation of incorrect isoforms was believed to be associated with one of the cancer hallmarks, genetic instability and mutation, summarized by Dougla Hanahan and Robert A. Weinberg [11, 12]. Moreover, growing evidence indicates that cancer-specific AS events possess potential applications in cancer therapy, serving as prognostic biomarkers and even therapeutic targets [9, 13].

Here, we repurposed and integrated multi-RNA-seq analysis among entries in the gastric tissue cohort from The Cancer Genome Atlas (TCGA) database to comprehensively analyze AS events. We systematically profiled the genome-wide AS events in GC and identified GC-related AS events. We also explored the potential biological function and underlying regulatory mechanisms of these specific cancer-related AS events in GC. Distinct clusters of GC-related AS events were identified, and the association between the distinct clusters and clinical and immune features was investigated. Finally, we performed survival analyses to identify the prognostic value of AS events.

## RESULTS

### Overview of AS events in the TCGA STAD cohort

**Figure 1:**
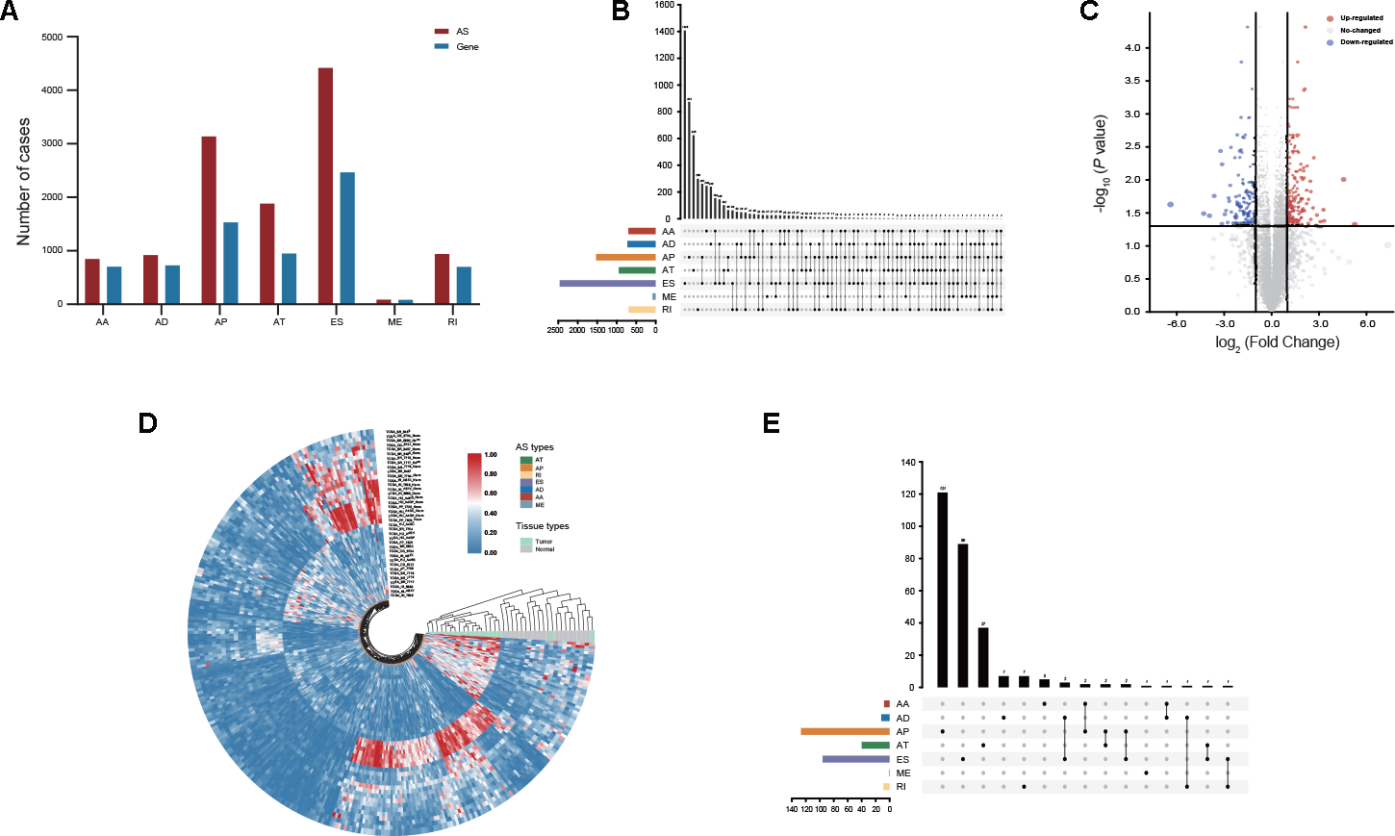
**Profiling of AS events identified in GC.** (**A**) The number of AS events and their parental genes derived from GC patients was counted based on the AS types. (**B**) Interactive analysis of seven types of AS events derived from GC patients is shown in an UpSet plot. (**C**) DEAS events between GC and adjacent normal tissues were visualized in a volcano plot. (**D**) Distinct DEAS events between GC and adjacent normal tissues were clustered and visualized in sector plots. (**E**) Interactive analysis of DEAS events between GC and adjacent normal tissues is shown in an UpSet plot.

The large-scale genome RNA-seq data of 305 stomach adenocarcinoma (STAD) patients from the TCGA database were analyzed, and a total of 12,225 AS events were identified from 5,199 genes. The included population comprised 846 alternate acceptor (AA) site events from 702 genes, 919 alternate donor (AD) site events from 727 genes, 3,137 alternate promoter (AP) events from 1,531 genes, 1,881 alternate terminator (AT) events from 948 genes, 4,417 exon skip (ES) events from 2,465 genes, 86 mutually exclusive exon (ME) events from 82 genes and 939 retained intron (RI) events from 698 genes (Figure 1A). According to the prioritization of proportion among AS events, ES events accounted for 36.13% of all AS events, whereas ME events accounted for 0.7% of all AS events. Intriguingly, a considerable proportion of genes contained two or more AS events, and 5 different AS events occurred in one single gene (Figure 1B).

### Identification of DEAS events in GC

To identify DEAS events in GC, we compared the percent spliced in (PSI) values of GC and adjacent normal tissues from the TCGA database. After screening, a total of 314 DEAS events from 280 genes were identified. Among these DEAS events, 175 DEAS events from 161 genes were upregulated, and 139 DEAS events from 127 genes were downregulated (Figure 1C, Supplementary Table 1). Intriguingly, GC and normal tissues were clearly separated into two discrete groups using unsupervised hierarchical clustering based on these DEAS events, but unfortunately, two GC tissues were misclassified as normal tissues (Figure 1D). There was an uneven distribution of splicing modes between DEAS events and all AS events (Figure 1B, 1E). Most genes had only one AS event, whereas some genes had up to two different splicing modes (Figure 1E).

**Figure 2:**
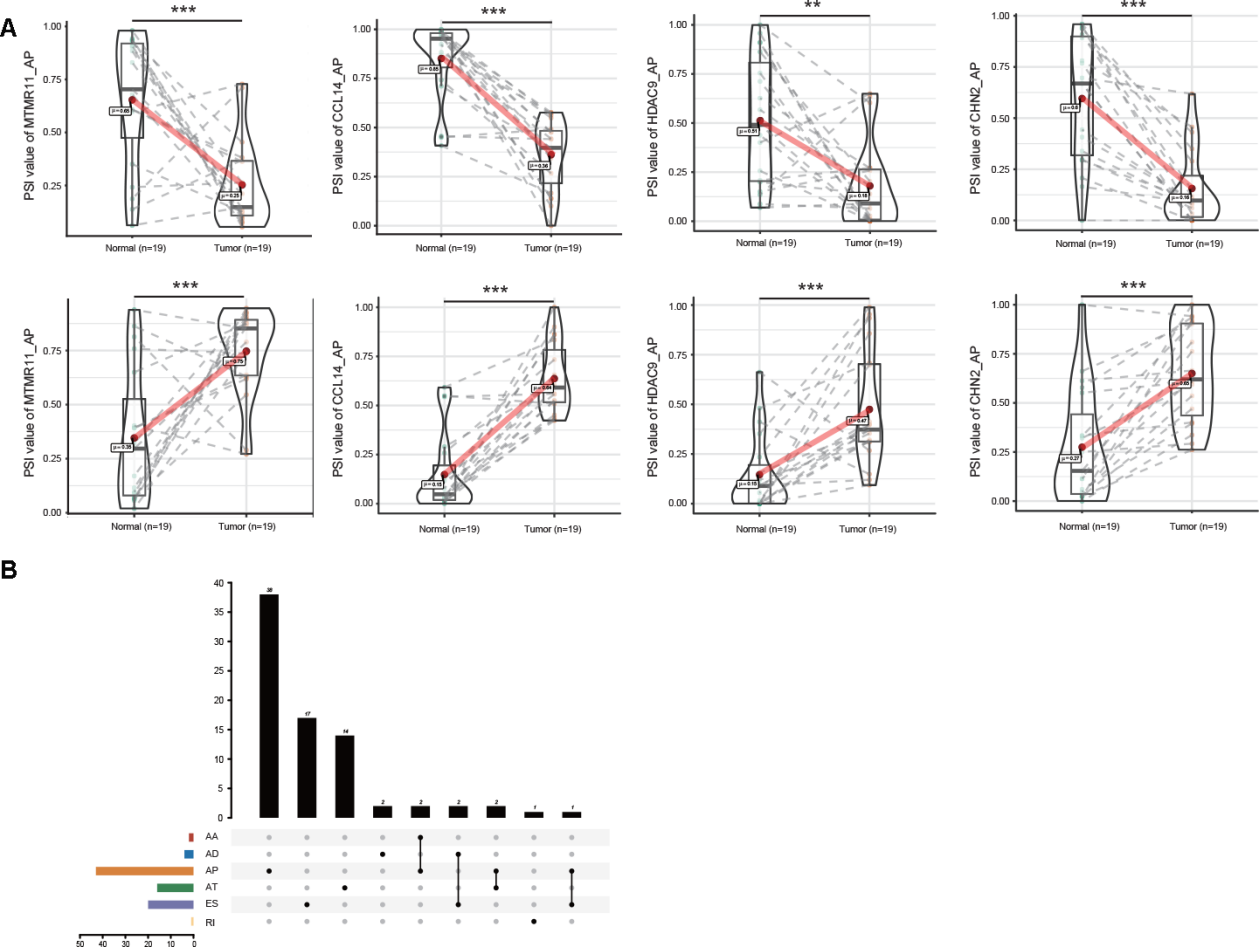
**Profiling of DEAS EVs identified in GC.** (**A**) The opposite trend of representative DEGs between GC and adjacent normal tissues is shown in violin plots. Some AS events of these representative DEGs were upregulated in GC tissues (upper). The same AS events of these representative DEGs were downregulated in GC tissues for the different splicing sites (lower). (**B**) Interactive analysis of DEGs between GC and adjacent normal tissues is shown in an UpSet plot.

Notably, some genes, such as MTMR11, CCL14, HDAC9 and CHN2, showed the opposite trends for the same splicing mode of the parental gene in GC and normal tissues (Figure 2A). Since aberrant AS events might directly affect the expression of parental genes, the relationship between DEAS events and differentially expressed genes (DEGs) was further assessed. By comparing 314 DEAS events from 280 parental genes between GC and normal tissues, a total of 79 DEGs involving 97 DEAS events were observed (Figure 2B, Supplementary Table 2). Spearman’s correlation analysis indicated that 69 of 97 (71.13%) DEAS events were significantly correlated with the expression of their parental gene (|R| ≥ 0.4 and adjusted P < 0.05). Moreover, almost half of these 69 DEAS events were AP events (44.93%) (Supplementary Figure 1, Supplementary Table 3).

### Enrichment and interaction analyses of DEAS events

**Figure 3:**
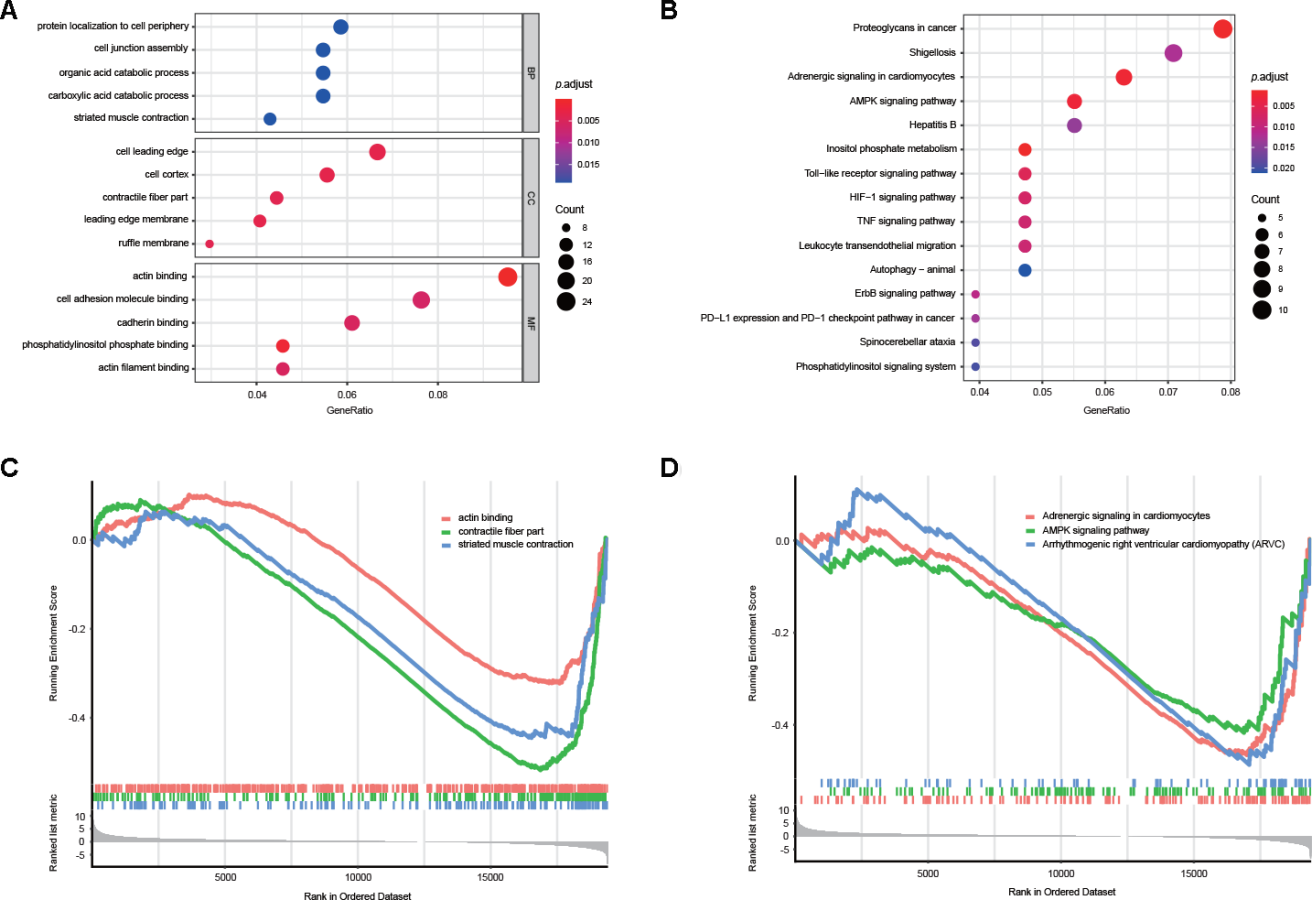
**Potential biological functions of DEAS events.** (**A**) GO analysis of DEAS events is shown in bubble plots. (**B**) KEGG analysis of DEAS events is shown in bubble plots. (**C**, **D**) GSEA of DEAS events.

The potential influence of DEAS events on the parental genes was then assessed with biological function enrichment analysis. The results revealed that certain GO categories, such as protein localization to the cell periphery, cell junction assembly, cell adhesion molecular binding and cadherin binding, were enriched in these 280 parental genes, which are closely associated with GC development (Figure 3A, Supplementary Table 4). Furthermore, certain vital KEGG pathways, such as proteoglycans in cancer, adrenergic signaling in cardiomyocytes, the AMPK signaling pathway, the HIF-1 signaling pathway and the TNF signaling pathway, were also enriched (Figure 3B, Supplementary Table 5). Consistent with these findings, gene set enrichment analysis (GSEA) revealed that DEAS events in GC were significantly enriched in actin binding, adrenergic signaling in cardiomyocytes and the AMPK signaling pathway (Figure 3C, 3D, Supplementary Tables 6, 7).

**Figure 4:**
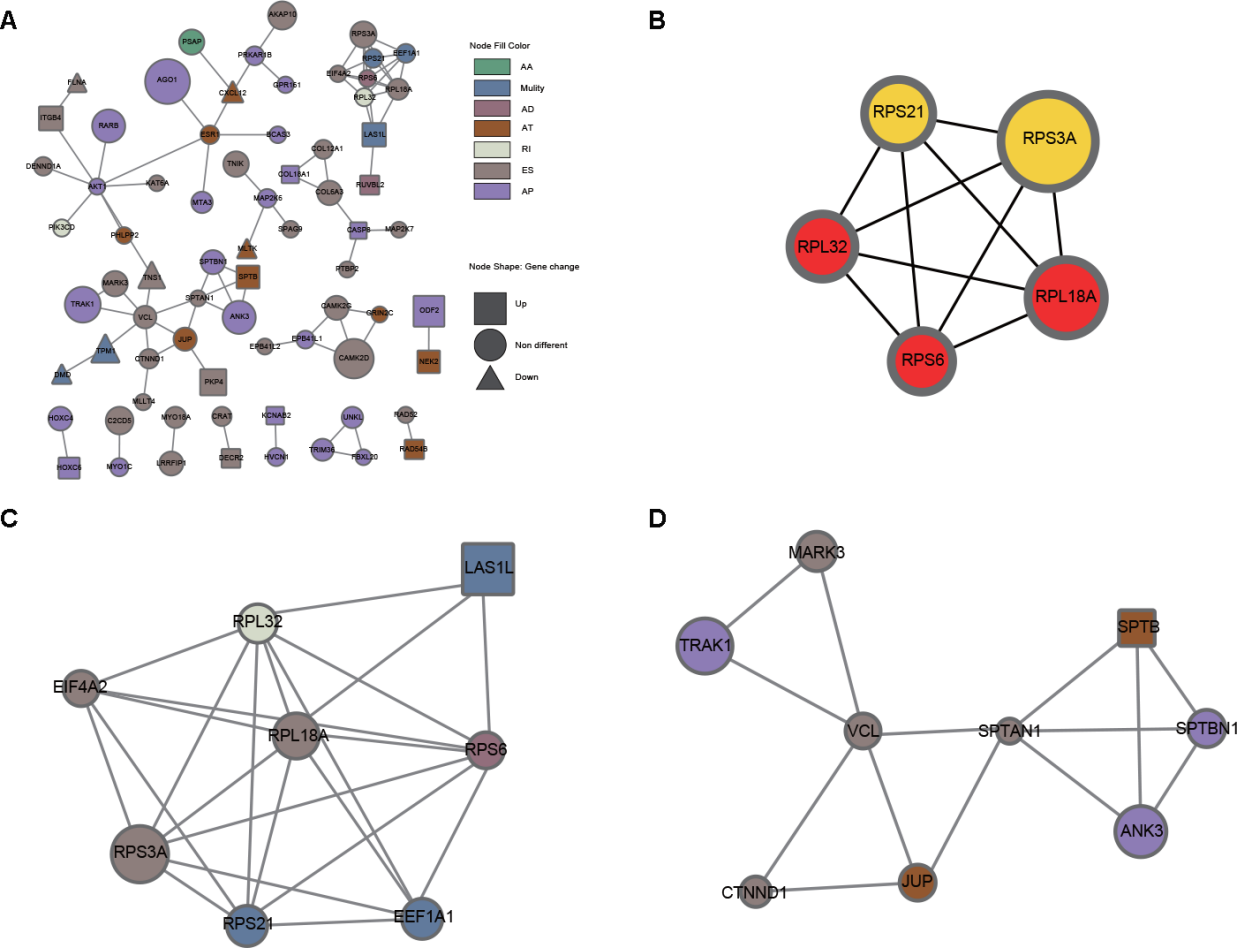
**PPI analysis of DEAS events.** (**A**) The interactome of 280 parental genes for 314 DEAS events is shown in the PPI network. (**B**) The interactome of the top 5 hub genes. (**C**) Module 1 was associated with ribosomes. (**D**) Module 2 was associated with adherens junctions and migration.

Since AS events could inevitably affect protein functions, a protein-protein interaction (PPI) network of the parental genes of DEAS events was constructed to reveal the potential influence of DEAS events at the protein level (Figure 4A). Moreover, hub genes and individual modules were further identified based on the PPI network. Among the PPI network, we found that the top 5 hub genes were RPS6, RPL32, RPL18A, RPS21 and RPS3A (Figure 4B). We also identified two individual modules (Figure 4C, 4D). Consistent with our results from the enrichment analysis, DEAS events in module 1 were enriched in polysomes (GO: 0005844) and ribosomes (KEGG: hsa03010), suggesting that the function of ribosomes might be frequently affected by DEAS events in GC (Supplementary Table 8). DEAS events in module 2 were enriched in adhesion junction (KEGG: hsa04520), leukocyte transendothelial migration (KEGG: hsa04670) and several other GO terms (Supplementary Table 9).

### Network of DEAS events and SFs

**Figure 5:**
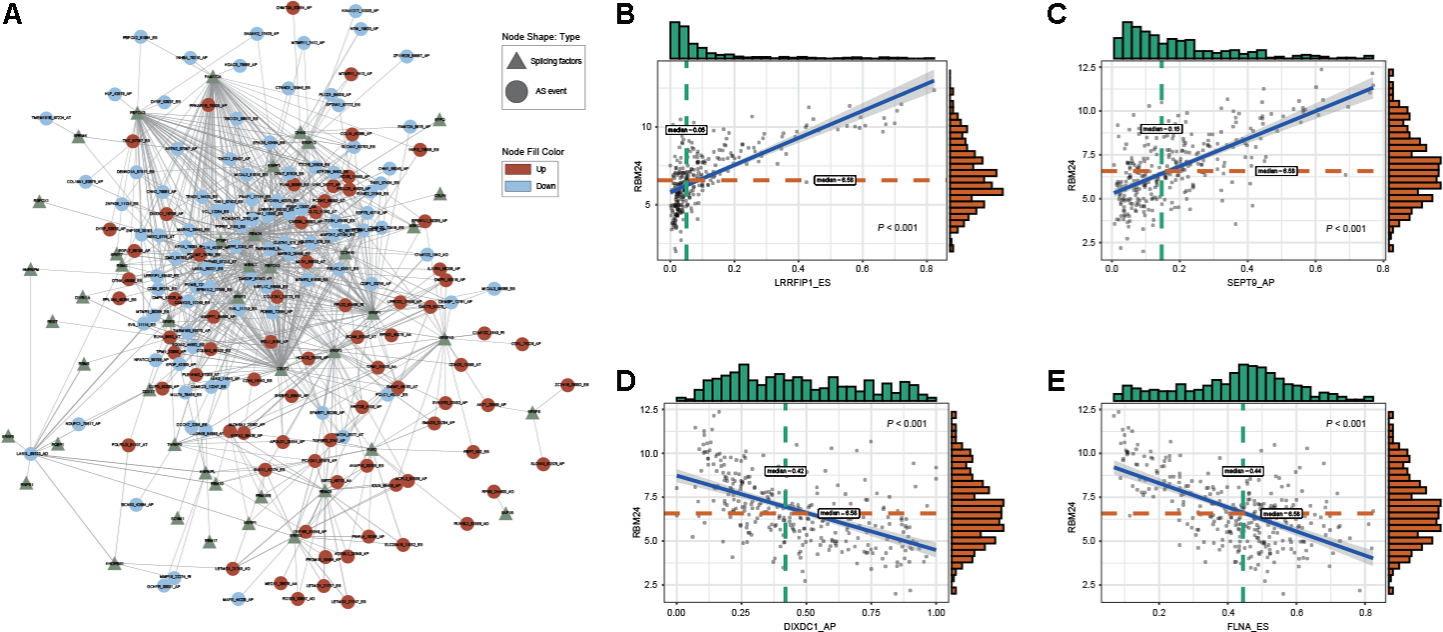
**Regulatory splicing correlation network in GC.** (**A**) The correlation of DEAS events with SFs is shown in network plots. (**B**, **C**) Representative positive correlations between DEAS events and SFs are shown in scatter plots. (**D**, **E**) Representative negative correlations between DEAS events and SFs are shown in scatter plots.

Alternative splicing patterns can be regulated by many types of RNA-binding proteins (RBPs), which are known as splicing factors (SFs) [14]. Among these special proteins, serine-arginine-rich splicing factor (SRSF) and heterogeneous nuclear ribonucleoprotein (hnRNP) are two well-known families of splicing regulatory factors [15]. To explore the potential regulatory relationship between DEAS events and SFs in GC, correlations between the PSI value of 314 DEAS events and the expression of SFs were analyzed in the TCGA STAD cohort. A total of 170 DEAS events were significantly associated with 44 SFs (|R| ≥ 0.4 and adjusted P < 0.05) (Figure 5A, Supplementary Table 10). In the SF-associated regulatory network, we observed that most SFs were significantly correlated with more than one DEAS event and that one DEAS event could be regulated by 13 different SFs, all of which characterize the comprehensive regulatory network of cooperative or competitive relationships between DEAS events and SFs. For example, RBM24 showed a positive correlation with LRRFIP1_ES and SEPT9_AP (Figure 5B, 5C). On the other hand, RBM24 showed a negative correlation with DIXDC1_AP and FLNA_ES (Figure 5D, 5E). Other representative correlations between DEAS events and SFs are presented in scatter plots (Supplementary Figure 2).

### DEAS-based cluster construction and correlations with molecular, clinical and immune features

**Figure 6:**
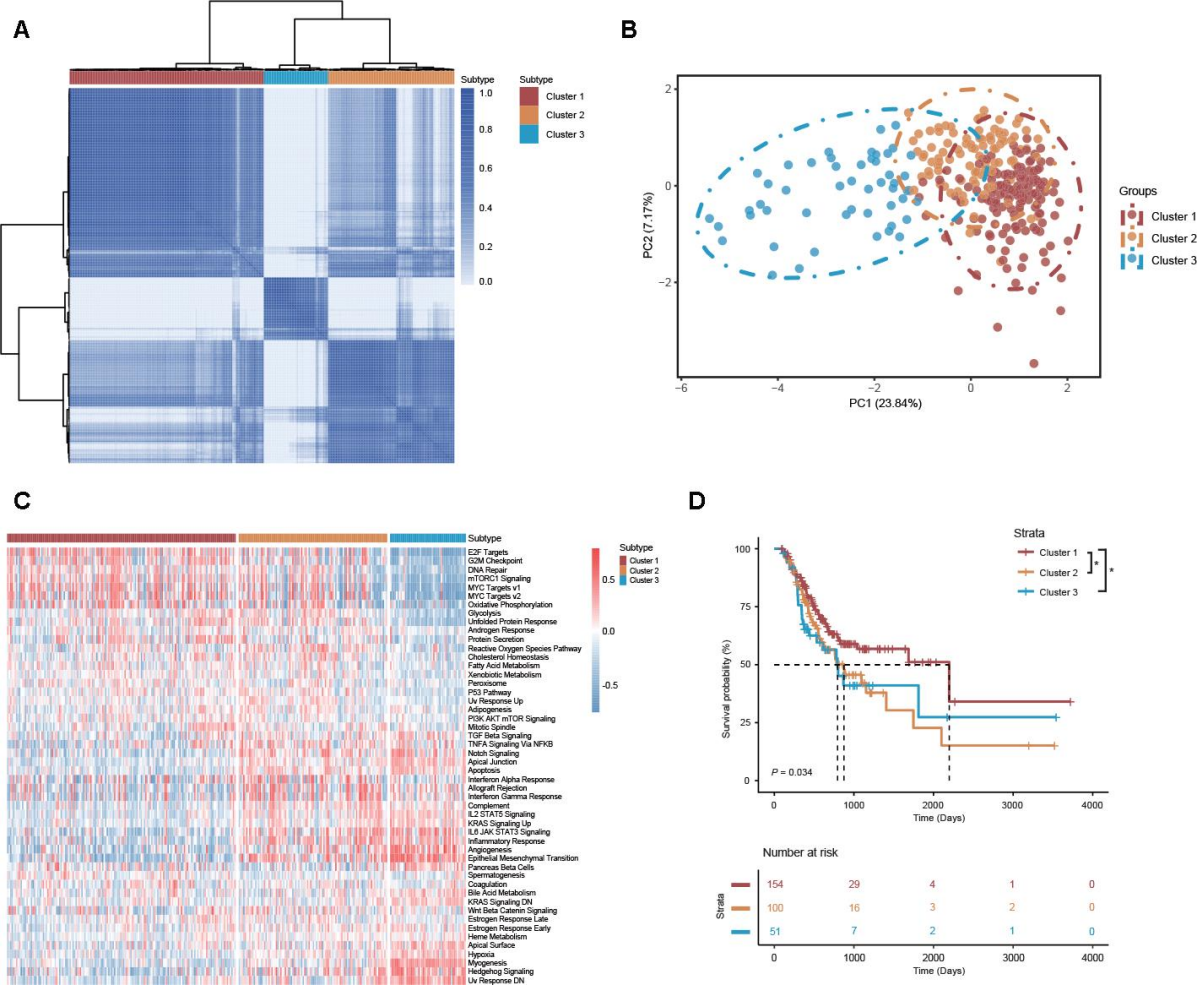
**DEAS-based clusters associated with molecular characteristics, cancer-specific signatures and prognosis.** (**A**) Consensus clustering analysis of 3 defined clusters was visualized in a matrix heatmap. (**B**) PCA of 3 distinct clusters is shown in scatter plots. (**C**) GSVA of cancer-specific signatures between DEAS-based clusters is shown in a cluster heatmap. (**D**) Kaplan-Meier survival analysis of patients within 3 distinct clusters of OS.

Because of molecular heterogeneity, GC can be clustered into molecular subtypes based on the distinct expression patterns of genes or proteins [4]. Here, consensus unsupervised analysis was performed on distinct clusters of GC patients according to the DEAS events that varied at the individual level. As shown in the consensus matrix heatmap, 3 clusters of GC patients were identified: cluster 1 (n = 154, 50.5%), cluster 2 (n = 100, 32.78%) and cluster 3 (n = 51, 16.72%) (Figure 6A). Consistent with the consensus clustering results, principal component analysis (PCA) also discerned significant differences among clusters 1, 2 and 3 (Figure 6B). In addition, a gene set variation analysis (GSVA) revealed a set of cancer-specific signatures that differed between the DEAS-based clusters (Figure 6C, Supplementary Table 11). This finding suggested that different clusters were associated with different biological processes, which further validated the reliability of our DEAS-based clusters.

**Figure 7:**
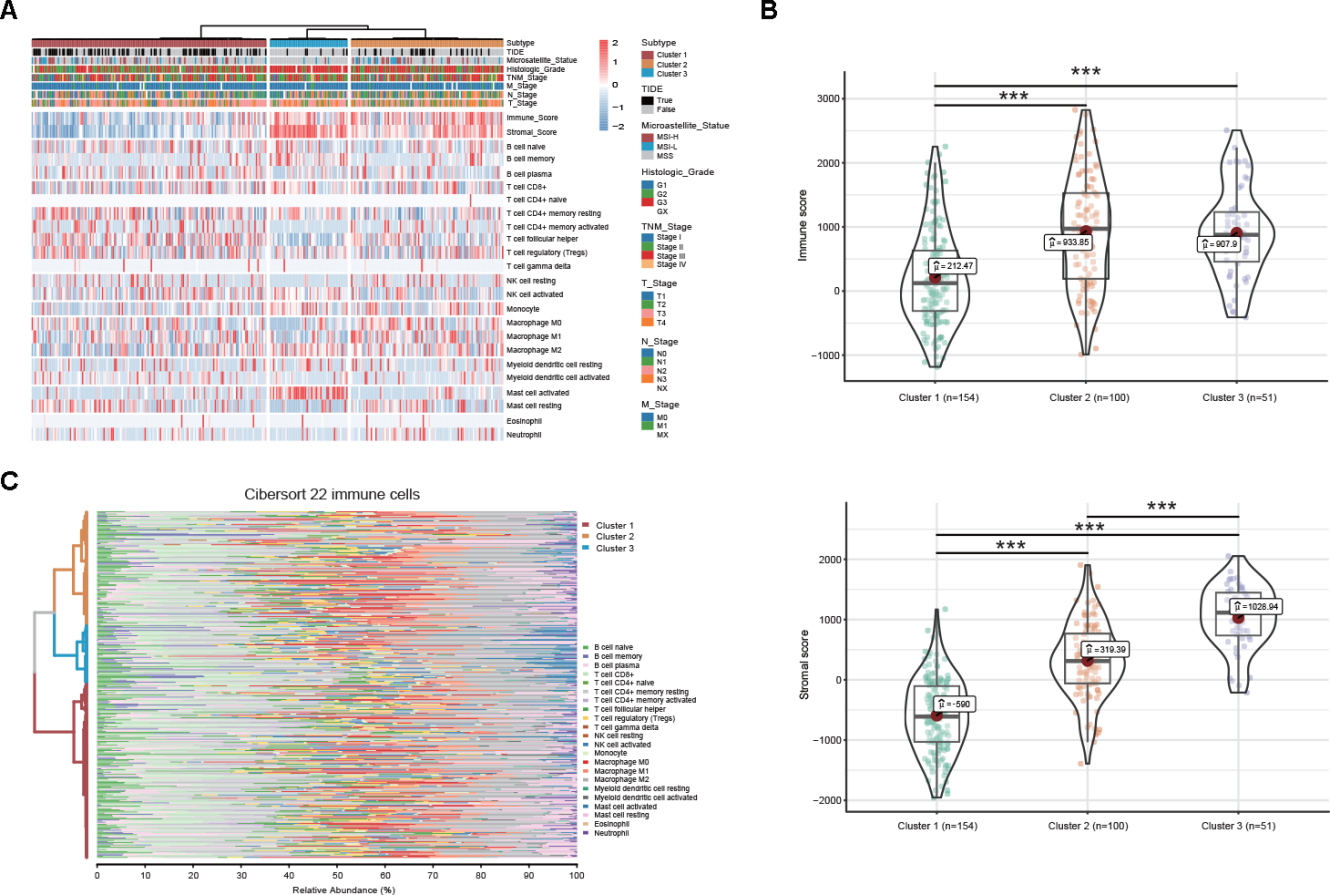
**DEAS-based clusters associated with clinicopathological characteristics and immune microenvironment features.** (**A**) A total of 305 DEAS events ordered by distinct clusters with annotations associated with clinicopathological characteristics and immune microenvironment features were visualized in a matrix heatmap. (**B**) Immune and stromal scores of each DEAS-based cluster. (**C**) Percentage matrix heatmap of immune cell infiltration in the tumor microenvironment between distinct clusters.

To study the clinical implications of the DEAS-based clusters, univariate survival analysis was conducted to assess the relationship between clusters and survival outcomes. We found that the survival outcome was not randomly distributed across different clusters (Figure 6D). GC patients belonging to cluster 1 had a better prognosis than those belonging to clusters 2 and 3 (Figure 6D). We also assessed the relationship between DEAS-based clusters and clinicopathological factors. The distributions of T stage, histologic grade and microsatellite status among the clusters were significantly different (Figure 7A, Supplementary Table 12).

To comprehensively characterize the immune features based on DEAS events, we investigated differences in the immune microenvironment and found that both the immune and stromal scores were significantly different between the DEAS-based clusters (Figure 7B, 7C). Notably, cluster 1, which was associated with a better prognosis, was associated with lower immune and stromal scores than clusters 2 and 3 (Figure 7B). Moreover, our studies of immune cell infiltration revealed that many types of immune cells were not randomly distributed across the different clusters (Figure 7A, 7C, Supplementary Table 12). Cluster 1 had a significantly lower proportion of non-immunotherapy-associated cells, such as activated mast cells, and a higher proportion of immunotherapy-associated cells, such as T cell follicular helper cells, compared with clusters 2 and 3 (Figure 7A, 7C Supplementary Table 12). These results provide further insight into immunotherapy, and the tumor immune dysfunction and exclusion (TIDE) algorithm was used to predict the likelihood of a response to immunotherapy. These results suggested that the DEAS-based clusters were correlated with different immune responses (Figure 7A, Supplementary Table 12). Patients in cluster 1 (50.64%, 78/154) presented a higher proportion of immunotherapy-associated cells and a lower proportion of stromal cells; thus, they might be more likely to respond to immunotherapy than patients in cluster 2 (27%, 27/100) and cluster 3 (13.72%, 7/51).

### Prognostic value of DEAS events in GC

**Figure 8:**
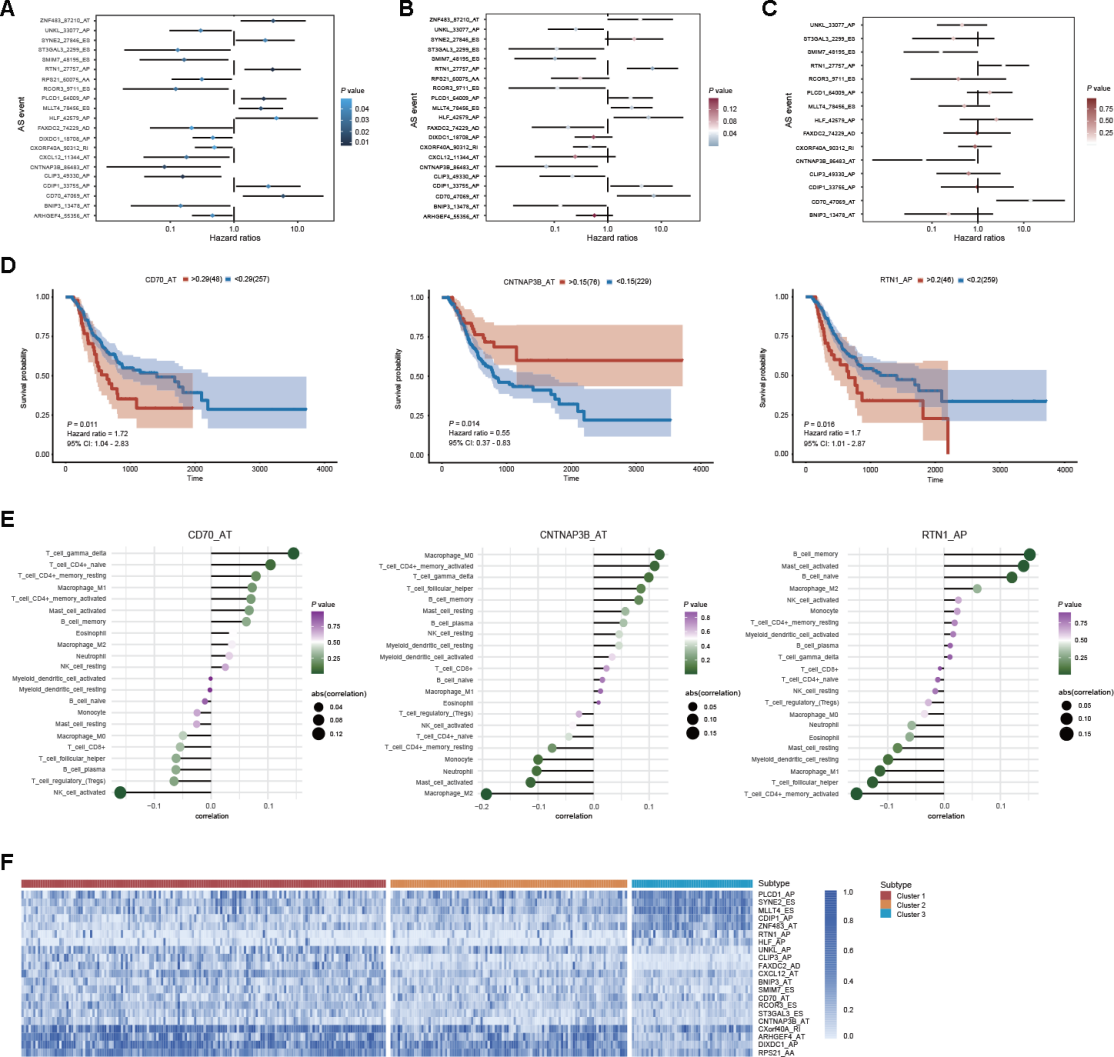
**Prognostic value of DEAS events in GC.** (**A**) Univariate analysis of DEAS events associated with OS is shown in forest plots of hazard ratios. (**B**) Univariate analysis of DEAS events associated with OS after adjusting for relative clinical covariates is shown in forest plots of hazard ratios. (**C**) Multivariate analysis of DEAS events associated with OS is shown in forest plots of hazard ratios. (**D**) Kaplan-Meier survival analysis of the independent prognostic DEAS events on OS. (**E**) Correlation analysis of the independent prognostic DEAS events and immune cells. (**F**) Differential expression of representative prognostic DEAS events between distinct clusters was visualized in a matrix heatmap.

To identify the potential clinical value of DEAS events, we investigated the underlying relationship between DEAS events and the prognosis of patients with GC. Among the 314 DEAS events, univariate Cox regression analysis revealed that 21 DEAS events were significantly associated with OS (Figure 8A). After adjusting for relative clinical covariates, 15 DEAS events were identified as independent prognostic factors (Figure 8B). Ultimately, in the multivariate Cox regression analysis, 3 of the 15 DEAS events were determined as independent prognostic indicators: CD70_AT, CNTNAP3B_AT and RTN1_AP (Figure 8C). As shown in Figure 8D, Kaplan-Meier analysis was conducted to assess the relationship between DEAS events and survival. Furthermore, each independent prognostic indicator DEAS event was found to be significantly associated with many types of immune cells (Figure 8E). Combined with the above DEAS-based clusters, we observed that DEAS events associated with good prognoses, such as CLIP3_AP, CNTNAP3B_AT and UNKL_AP, were highly expressed in cluster 1. In contrast, DEAS events associated with poor prognoses, such as PLCD1_AP, MLLT4_ES and ZNF483_AT, were highly expressed in clusters 2 and 3 (Figure 8F).

## DISCUSSION

AS, as a crucial posttranscriptional modification, allows cells to generate multiple RNA and protein isoforms with distinct structural, regulatory and functional properties [8, 9]. It has been determined that abnormal AS events contribute to numerous diseases, including several types of cancer [10, 11]. Accumulating evidence has also proven that the specific dysregulation of AS events plays crucial roles in the initiation and progression of GC [16]. For instance, CD44, MUTYH and WNT2B splice variants participate in the metastasis, carcinogenesis and development of GC [17–19]. Because of the limited sample sizes of one or a few specific AS events in previous studies, the exploration of AS events in GC is far from comprehensive. Hence, we systematically profiled AS events in a large-scale GC cohort to elucidate the landscape of AS events in GC.

Through a rigorous filter, a total of 12,225 AS events of 5,199 genes were identified in 305 GC patients, indicating that AS is a common posttranscriptional modification in GC. Moreover, 314 DEAS events were detected from 280 genes among the GC and normal tissues. The correlation analysis indicated that most of the DEAS events were significantly correlated with the expression of their parental gene, consistent with the hypothesis that AS acts as an important segment of the posttranscriptional process and alters gene expression [20]. All the above experimentally validated splice variants were also identified by our procedure, suggesting that our results are reliable and that the DEAS events identified in our study are ubiquitous in GC. In addition, we found that GC shares some common DEAS events with those in colorectal and head-neck squamous cell cancers, indicating that certain AS events are common in cancer tumorigenesis and development [21, 22].

To explore the potential mechanism of DEAS events in GC, we performed functional enrichment analysis and generated a PPI network of their parental genes. In the functional enrichment analysis, the parental genes were mainly enriched in pathways related to the cytoplasm and extracellular matrix, such as “cell junction organization and assembly”, “cell adhesion molecular binding and cadherin binding” and “AMPK signaling pathway”. The cytoplasm is the major location of AMPK, and the AMPK signaling pathway is considered a vital factor in the development and treatment of GC [23–26]. Abnormal cell adhesion and cell junctions are also associated with the progression and metastasis of GC, which further supports the accuracy and reliability of our enrichment analysis [27–29]. Correspondingly, the DEAS events influenced the above potential pathophysiological processes in GC. By constructing the interaction network of the parental genes, we found that most were correlated with each other. Two individual modules were highlighted in the PPI network, suggesting potential molecular complexes. Of note, all top 5 hub genes belonging to the ribosomal protein family comprised one individual module, which implies that the functions of ribosomes might be affected by DEAS events in GC.

SFs have been reported to participate in the precise regulation of RNA splicing by binding to specific RNA sequences [30]. Thus, we performed an integrated analysis of SFs and DEAS events to address the underlying mechanism of the splicing pathway in GC. The splicing correlation network showed distinguished interactions between DEAS events and SFs. Of note, we found that the same SF might play dual roles in the regulation of AS events and that the same AS event could be synergistically or antagonistically regulated by different SFs. This suggests that complex regulatory mechanisms may influence SFs and AS events. Indeed, the real regulatory mechanisms involved in RNA splicing could be more sophisticated than we revealed in this study. The relationship between DEAS events and SFs should be considered a dynamic interaction network instead of a simple “one to one” pattern.

Increasing evidence has demonstrated that AS events play an indispensable role in immune microenvironment formation [13]. Actual identification of the distinct patterns of AS events in the tumor microenvironment will contribute to GC therapy. Here, we revealed three distinct DEAS-based patterns among 314 DEAS events, and these three patterns presented distinct molecular, clinical and immune features. We found that most of the cancer hallmark pathways were significantly different between the 3 DEAS-based clusters and that pathogenetic pathways were broadly different regarding tumor biology. This further indicates that the DEAS-based clusters identified herein are reliable. According to our results, GC patients belonging to cluster 1 were significantly associated with a low histologic grade, a low T stage and a favorable survival outcome.

Moreover, cluster 1 was characterized by high tumor purity and was significantly associated with low immune and stromal scores. Consistent with our results, previous studies have also determined that low tumor purity is associated with a poor prognosis and an enhanced immune phenotype [31–33]. Referring to the amount of immune cell infiltration, the relative proportion of M2 macrophages and neutrophils is substantially inversely correlated with tumor purity [31–33]. Consistent with this finding, we found that the relative proportion of multiple types of immune cells was significantly low in cluster 1. According to the TIDE algorithm, the low proportion of immune cell types indicated that tumor purity was an essential factor of immunotherapy in GC. In addition, cluster 1 presented a higher proportion of immunotherapy-associated cells and a lower proportion of stromal cells, which was beneficial for immunotherapy. GC patients belonging to cluster 1 might obtain a better response than patients in other clusters. These findings suggest that the DEAS events in GC confer essential biological and clinical implications.

Due to the potential significance of AS events in tumor biology, their clinical relevance in GC was further assessed by survival analysis. A total of 21 DEAS events were found to be associated with OS. Among these survival-associated DEAS events, some genes were determined to play crucial roles in tumor biology. For instance, PLCD1 inhibits tumor formation in breast cancer by inducing apoptosis [34]. HLF transactivates c-Jun to promote tumor-initiating cell generation in hepatocellular carcinoma [35]. The inactivation of BNIP3 likely plays a vital role in the progression of colorectal cancer and GC [36]. Moreover, 15 of 21 survival-associated DEAS events also showed a significant association with OS, even after adjusting for multiple clinical factors, including sex, age and TNM stage. Finally, 3 of 21 survival-associated DEAS events were revealed as independent prognostic factors, and these 3 DEAS events performed well in stratifying GC patients into groups based on survival through Kaplan-Meier survival curves. In addition, each of these 3 DEAS events showed a significant association with several types of immune cells. The AS landscape profile in GC was mapped, thus providing an overview of this research, and the findings elucidated the functional roles of the splicing network in GC. Unfortunately, a limited number of validation experiments were performed; however, we intend to perform additional validation experiments in the future.

Our study depicted a comprehensive landscape of AS events in GC patients, and the implementation of strict criteria ensured the identification of abnormal AS events related to GC. Our functional analysis indicated that 314 DEAS events identified in our study might play essential roles in the development of GC. The SF-AS regulatory network further clarified the underlying mechanism of the splicing-associated pathway. Moreover, based on the DEAS events, the comprehensive clustering analysis of GC revealed the intrinsic relevance of molecular alterations and immune features, which indicated the value of AS events in predicting the clinical outcomes of GC patients. In addition, survival-related DEAS events might not only serve as prognostic indicators and therapeutic targets for GC patients but also help to decipher the mechanism of AS events in GC oncogenesis.

## MATERIALS AND METHODS

### Data acquisition and curation

Patients who met the following criteria were included in the TCGA GC cohort: (1) patients who were histologically confirmed as having primary GC; (2) patients with RNA-seq data; (3) patients with alternative RNA splicing data; (4) patients with detailed clinicopathological and follow-up information, including sex, age, race, TNM stage, histologic grade, microsatellite status and overall survival (OS) status; and (5) patients with an OS time of over 90 days. The corresponding RNA-seq data and clinical information of the GC cohort were downloaded from the TCGA data portal using the “GDCRNATools” package [37]. The corresponding alternative RNA splicing data of the GC cohort were downloaded from the TCGA SpliceSeq dataset [38]. Splicing events in the dataset were divided into seven categories: ES, RI, AP, AT, AD, AA and ME. Each splicing event was quantified by the PSI value [39], which ranges from 0 to 1 and represents the ratio of normalized read counts to indicate the inclusion of a transcript element over the total normalized reads for that event. To generate a reliable set of AS events, we implemented a series of stringent filters, which included “percentage of samples with a PSI value ≥ 75%”, “average PSI value ≥ 0.05” and “standard deviation of the PSI value ≥ 0.1”. Only AS events meeting the above criteria were included in the present analysis. Moreover, we filled in the missing PSI values using the k nearest neighbors algorithm [40].

### DEAS event identification and functional analysis

To identify the DEAS events in GC, we applied the paired sample *t*-test to compare the PSI values between tumor tissues and matched adjacent normal tissues, and the *P*-value was adjusted by the Benjamini-Hochberg (BH) method. AS events with an absolute value of log2-fold change ≥ 1 and an adjusted *P*-value < 0.05 were considered statistically significant. Then, we subjected the parental genes of the DEAS events to a biological function enrichment analysis, which was performed with the “clusterProfiler” package [41]. Gene Ontology (GO) terms, including “biological process (BP)”, “cellular component (CC)” and “molecular function (MF)”, and Kyoto Encyclopedia of Genes and Genomes (KEGG) pathways with adjusted *P* values < 0.05 were selected for further analysis. In addition, the parental genes of these DEAS events were mapped to coding proteins, and the PPIs between these coding proteins were downloaded from the Search Tool for the Retrieval of Interacting Genes/Proteins (STRING) database. A minimum required interaction score of 0.9 was used to identify reliable interaction results. The PPI network was further visualized with Cytoscape [42]. Hub genes and specific modules of the PPI network were identified by CytoHubba and the Molecular Complex Detection (MCODE) plug-in of Cytoscape [43, 44].

### SFs and splicing correlation network

A total of 78 genes that participated in the process of alternative RNA splicing (GO: 0000380) were obtained from the Molecular Signatures Database (MSigDB) [45]. The raw count value of these splicing genes was then extracted from the RNA-seq data and normalized by trimmed means of M (TNM) values [46]. We implemented the “voom” method to further transform the raw counts [47]. Spearman’s correlation analysis was conducted to explore the correlations between the expression of the splicing genes and the PSI values of the DEAS events. The *P*-value was adjusted by the BH method, and a correlation coefficient ≥ 0.4 and an adjusted *P*-value < 0.05 were considered statistically significant. The correlation network of the DEAS events and splicing genes was visualized with Cytoscape [42].

### Cluster analysis and correlation with molecular, clinical and immune features

Based on the identified DEAS events (n = 314), unsupervised clustering of the TCGA STAD cohort was performed via hierarchical consensus clustering with the “ConsensusClusterPlus” package [48]. Associations between clusters and clinicopathological variables (pathological T stage, pathological N stage, pathological M stage, pathological TNM stage, histologic grade and microsatellite status), survival status (OS), immune features (immune score, stromal score and immune cell infiltration) and immunotherapy responses between clusters were analyzed. Immune and stromal scores were calculated based on the ESTIMATE algorithm [49]. Immune cell infiltration was analyzed by CIBERSORT [50]. The TIDE algorithm was used to predict the clinical response to immunotherapy [51, 52]. GSVA was performed by the “clusterProfiler” package to investigate differences in the biological processes between the distinct DEAS-based clusters, and those with an adjusted *P*-value < 0.05 were considered statistically significant [41, 53].

### Survival analysis

To determine the survival-associated DEAS events in GC, we performed a univariate Cox proportional hazards regression analysis to estimate the PSI value of DEAS events with OS. DEAS events with a *P*-value < 0.05 in the univariate Cox regression analysis were selected as candidate prognostic AS events. To investigate whether these candidate prognostic AS events were independent clinical indicators, a multivariate Cox regression analysis was then applied and adjusted for the following clinically relevant covariates: sex, age, pathological T stage, pathological N stage, pathological M stage, pathological TNM stage, histologic grade and microsatellite status. Furthermore, based on the PSI value of each independent prognostic AS event, patients were divided into low-risk and high-risk subgroups according to the optimal cutoff value, which was determined by the “survminer” package [54]. Kaplan-Meier survival analyses with log-rank tests were performed to demonstrate the survival probability variation over time between the low-risk and high-risk subgroups, and *P*-values < 0.05 were considered statistically significant.

## Supplementary Material

Supplementary Figures

Supplementary Table 1

Supplementary Table 2

Supplementary Table 3

Supplementary Table 4

Supplementary Table 5

Supplementary Table 6

Supplementary Table 7

Supplementary Table 8

Supplementary Table 9

Supplementary Table 10

Supplementary Table 11

Supplementary Table 12
