# Supplementary material for: The landscape of alternative splicing reveals novel events associated with tumorigenesis and the immune microenvironment in gastric cancer
Source: Aging (Albany NY). 2021 Jan 10;13(3):4317–34. doi: 10.18632/aging.202393 (PMC7906195; doi:10.18632/aging.202393)
Supplement: Supplementary Figures [file aging-13-202393-s001.pdf]

SUPPLEMENTARY FIGURES

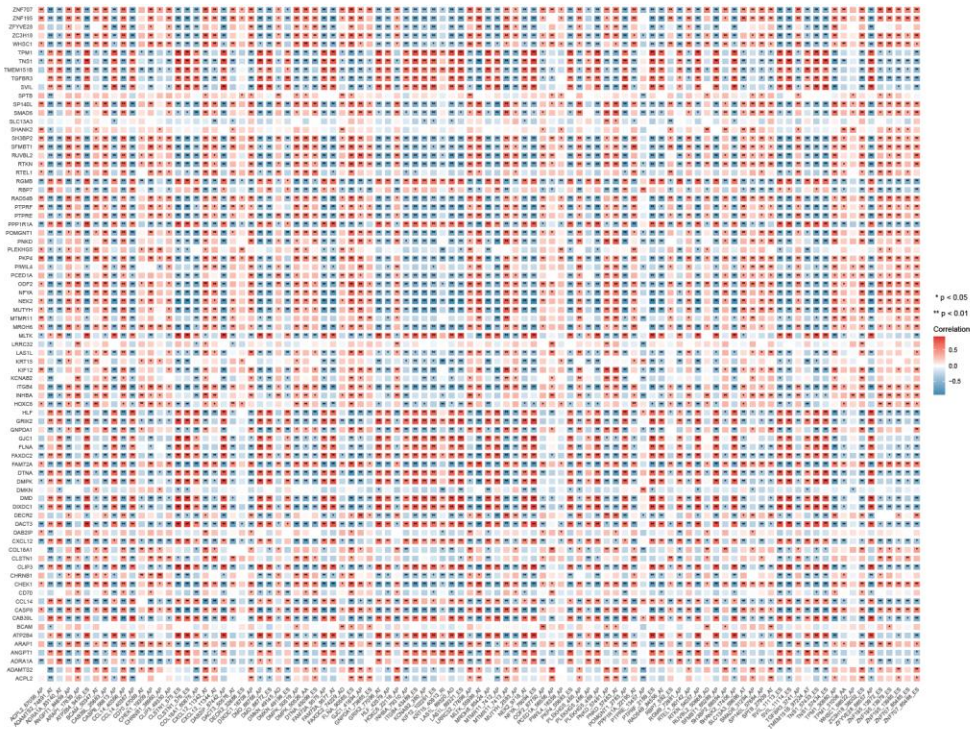

Supplementary Figure 1. Correlation heatmap of DEAS events and their parental genes.

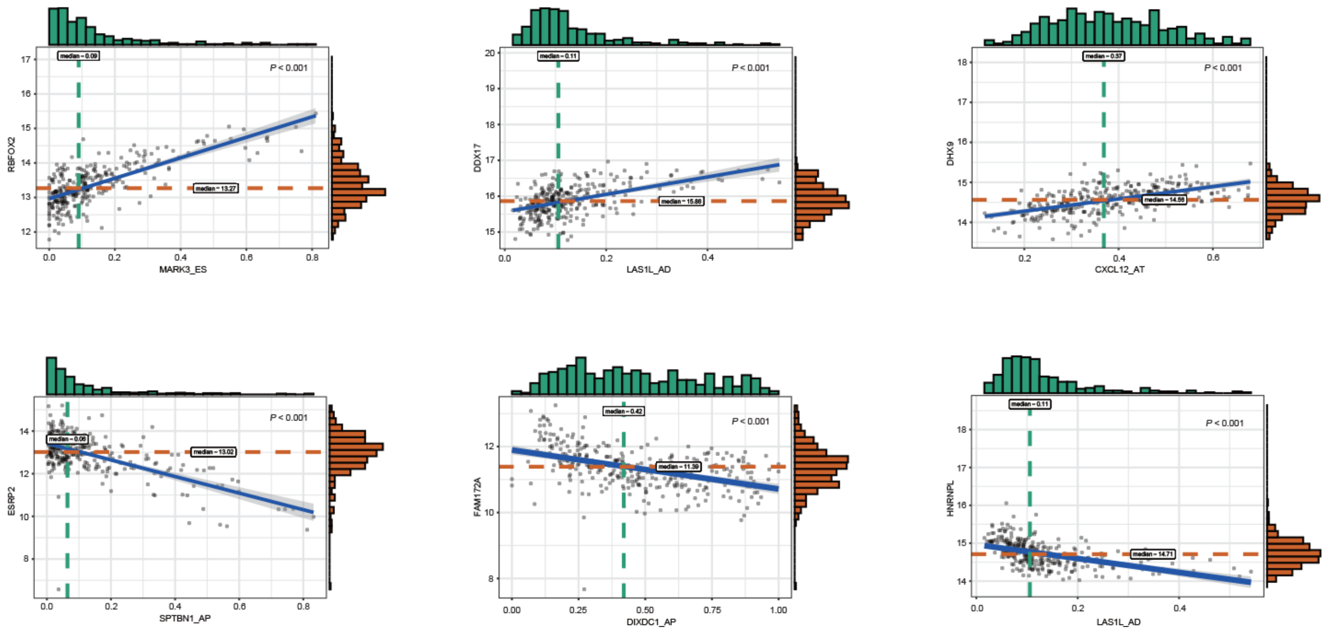

Supplementary Figure 2. Representative correlation between DEAS events and SFs.
