# Supplementary material for: The landscape of alternative splicing reveals novel events associated with tumorigenesis and the immune microenvironment in gastric cancer
Source: Aging (Albany NY). 2021 Jan 10;13(3):4317–34. doi: 10.18632/aging.202393 (PMC7906195; doi:10.18632/aging.202393)
Supplement: Supplementary Table 5 [file aging-13-202393-s006.pdf]

SUPPLEMENTARY TABLE

Supplementary Table 5. KEGG enrichment analysis of DEAS events.

KEGG ID	Description	GeneRatio	BgRatio	Adjusted P-value	gene ID
hsa00562	Inositol phosphate metabolism	6/127	74/8032	0.0010682	MTMR1/MTMR3/MTMR4/ALDH6A1/PIK3CD/PLCD1
hsa05205	Proteoglycans in cancer	10/127	204/8032	0.0014152	CD44/ESR1/WNT2B/RPS6/AKT1/CAMK2D/CAMK2G/ANK3/PIK3CD/FLNA
hsa04261	Adrenergic signaling in cardiomyocytes	8/127	149/8032	0.0024234	ATP2B4/ADRA1A/PPP1R1A/TPM1/AKT1/CAMK2D/CAMK2G/CACNA2D4
hsa04152	AMPK signaling pathway	7/127	120/8032	0.0028412	TBC1D1/CAB39L/LEPR/ADRA1A/AKT1/SCD5/PIK3CD
hsa04620	Toll-like receptor signaling pathway	6/127	104/8032	0.0059612	CASP8/MAP2K7/MAP2K6/AKT1/TIRAP/PIK3CD
hsa04066	HIF-1 signaling pathway	6/127	109/8032	0.0074647	RPS6/AKT1/CAMK2D/CAMK2G/ANGPT1/PIK3CD
hsa04668	TNF signaling pathway	6/127	112/8032	0.0084888	CASP8/MAP2K7/MAP2K6/DAB2IP/AKT1/PIK3CD
hsa04670	Leukocyte transendothelial migration	6/127	113/8032	0.0088515	CD99/VCL/CXCL12/AFDN/PIK3CD/CTNND1
hsa04012	ErbB signaling pathway	5/127	85/8032	0.0109392	MAP2K7/AKT1/CAMK2D/CAMK2G/PIK3CD
hsa05131	Shigellosis	9/127	236/8032	0.0123094	CD44/VCL/AKT1/WIPI2/PIK3CD/BNIP3/SEPTIN9/PLCD1/FNBP1
hsa05235	PD-L1 expression and PD-1 checkpoint pathway in cancer	5/127	89/8032	0.0131715	NFATC2/MAP2K6/AKT1/TIRAP/PIK3CD
hsa05161	Hepatitis B	7/127	162/8032	0.0141447	CASP8/MAP2K7/NFATC2/MAP2K6/AKT1/TIRAP/PIK3CD
hsa05017	Spinocerebellar ataxia	5/127	98/8032	0.0192755	NFYA/AKT1/WIPI2/GRIN2C/PIK3CD
hsa04070	Phosphatidylinositol signaling system	5/127	99/8032	0.0200512	MTMR1/MTMR3/MTMR4/PIK3CD/PLCD1
hsa04140	Autophagy - animal	6/127	137/8032	0.0212426	MTMR3/MTMR4/AKT1/WIPI2/PIK3CD/BNIP3
hsa04720	Long-term potentiation	4/127	67/8032	0.0212478	PPP1R1A/CAMK2D/CAMK2G/GRIN2C
hsa04664	Fc epsilon RI signaling pathway	4/127	68/8032	0.0223072	MAP2K7/MAP2K6/AKT1/PIK3CD
hsa05152	Tuberculosis	7/127	180/8032	0.0237873	NFYA/CASP8/LSP1/AKT1/CAMK2D/CAMK2G/TIRAP
hsa04625	C-type lectin receptor signaling pathway	5/127	104/8032	0.0242368	CASP8/NFATC2/LSP1/AKT1/PIK3CD
hsa04520	Adherens junction	4/127	71/8032	0.0256768	VCL/AFDN/PTPRF/CTNND1
hsa05214	Glioma	4/127	75/8032	0.0306229	AKT1/CAMK2D/CAMK2G/PIK3CD
hsa05412	Arrhythmogenic right ventricular cardiomyopathy (ARVC)	4/127	77/8032	0.0332926	ITGB4/CACNA2D4/JUP/DMD
hsa04724	Glutamatergic synapse	5/127	114/8032	0.0342061	SLC38A1/GLUL/GRIN2C/SHANK2/GRIK2
hsa04510	Focal adhesion	7/127	199/8032	0.0380996	VCL/TNC/ITGB4/AKT1/COL6A3/PIK3CD/FLNA
hsa03010	Ribosome	6/127	158/8032	0.0389509	MRPL22/RPL18A/RPS6/RPL32/RPS3A/RPS21
hsa04722	Neurotrophin signaling pathway	5/127	119/8032	0.0400213	MAP2K7/AKT1/CAMK2D/CAMK2G/PIK3CD
hsa05135	Yersinia infection	5/127	120/8032	0.0412523	MAP2K7/NFATC2/MAP2K6/AKT1/PIK3CD
